# Ultralow-temperature device dedicated to soft X-ray magnetic circular dichroism experiments

**DOI:** 10.1107/S1600577518012717

**Published:** 2018-10-19

**Authors:** J.-P. Kappler, E. Otero, W. Li, L. Joly, G. Schmerber, B. Muller, F. Scheurer, F. Leduc, B. Gobaut, L. Poggini, G. Serrano, F. Choueikani, E. Lhotel, A. Cornia, R. Sessoli, M. Mannini, M.-A. Arrio, Ph. Sainctavit, P. Ohresser

**Affiliations:** a Synchrotron SOLEIL, L’Orme des Merisiers, BP 48, 91192 Gif-sur-Yvette, France; b Université de Strasbourg, CNRS, Institut de Physique et Chimie des Matériaux de Strasbourg, UMR 7504, 23 rue du Loess, 67034 Strasbourg, France; c Department of Chemistry ‘U. Schiff’ and INSTM RU, University of Firenze, Via della Lastruccia n. 3, 50019 Sesto Fiorentino (FI), Italy; d Institut Néel, 25 rue des Martyrs, 38042 Grenoble, France; e Department of Chemical and Geological Sciences and INSTM RU, University of Modena and Reggio Emilia, via G. Campi 103, I-41125 Modena, Italy; f Institut de Minéralogie, de Physique des Matériaux et de Cosmochimie, CNRS, Sorbonne Université, IRD, MNHN, UMR 7590, 4 place Jussieu, 75252 Paris Cedex 05, France

**Keywords:** ultralow-temperature, soft X-rays, XMCD, magnetism

## Abstract

A new ultralow-temperature setup dedicated to soft X-ray absorption spectroscopy and X-ray magnetic circular dichroism (XMCD) experiments is described. Two experiments, performed on the DEIMOS beamline (SOLEIL synchrotron), demonstrate the outstanding performance of this new platform in terms of the lowest achievable temperature under X-ray irradiation (*T* = 220 mK), the precision in controlling the temperature during measurements as well as the speed of the cooling-down and warming-up procedures. Moreover, owing to the new design of the setup, the eddy-current power is strongly reduced, allowing fast scanning of the magnetic field in XMCD experiments; these performances lead to a powerful device for X-ray spectroscopies on synchrotron-radiation beamlines facilities.

## Abstract 2

A new ultralow-temperature setup dedicated to soft X-ray absorption spectroscopy and X-ray magnetic circular dichroism experiments is described. Two experiments, performed on the DEIMOS beamline (SOLEIL synchrotron), demonstrate the outstanding performances of this new platform, particularly with regard to the lowest achievable temperature under X-ray irradiation (*T* = 220 mK).

## 1. Introduction   

The synchrotron-based X-ray absorption spectroscopies, X-ray magnetic circular dichroism (XMCD) and linear dichroism (XLD), are particularly useful techniques in physics, chemistry and materials science because of their chemical selectivity and high sensitivity. For instance, they allow investigation of diluted elements in the first nanometres of bulk samples down to a concentration of few hundred p.p.m., and collections of single molecules or atoms on surfaces (Van der Laan & Figueroa, 2014 ▸; Stöhr *et al.*, 1998 ▸). Moreover, they provide information on both static and dynamic magnetic properties, including magnetic polarization and anisotropy, spin and orbital contributions to magnetic moments (Thole *et al.*, 1992 ▸; Carra *et al.*, 1993 ▸), magnetic coupling between spins localized on different elements (Joly *et al.*, 2017 ▸; Ohresser *et al.*, 2005 ▸) and magnetic bistability (Gambardella *et al.*, 2002 ▸; Margheriti *et al.*, 2010 ▸). A dichroic spectrum is obtained from the difference between two X-ray absorption spectra (XAS) recorded with different light polarizations (circular left and right for XMCD, linear vertical and horizontal for XLD). When measuring XMCD, an external magnetic field is generally used to control the magnetic state of the sample. In the case of ferromagnetic samples, with large enough remnant magnetization and small enough coercive fields, XMCD experiments can also be performed in low external magnetic fields or in the remnant state. In order to reach the high sensitivity of this technique, it is crucial that all parameters remain stable over the entire duration of the measurements. This implies a highly reliable photon source, ensuring a constant polarization rate and photon flux; a very steady optical pathway, ensuring high-energy stability; and a constant sample environment in terms of temperature and magnetic field. The DEIMOS beamline, located on a medium straight section (I07-m) of the storage ring at the French Synchrotron SOLEIL (Source Optimisée de Lumière à Energie Intermédiaire du LURE, https://www.synchrotron-soleil.fr/), has been conceived specifically to best fulfill these requirements (Ohresser *et al.*, 2014 ▸).

The accessible energy range on the DEIMOS beamline, with the aforementioned four polarizations, ranges from 350 eV to 2500 eV. This covers the *L* absorption edges of 3*d* and 4*d* transition elements, the *M*-edges of rare earth elements and the *K*-edge of nitrogen, oxygen and sulfur atoms. All these elements are of particular interest in the field of molecular magnetism and, more generally, in magnetism of nano objects.

Ultralow-temperature (ULT, *T* < 1 K) is of primary importance when dealing with new states of matter and studies of magnetic phenomena such as spin transitions, magnetic ordering, relaxation of metastable states, superconductivity, Kondo effect, *etc*. When the thermal energy is smaller than the energy difference between the ground state and the first excited levels, the ground state properties can be revealed.

Among cryogenic fluids, liquid ^4^He and ^3^He allow us to reach a limit temperature, *T*_lim_, of about 1 K and 0.3 K, respectively, at sub-atmospheric pressure. To reach even lower temperatures, one takes advantage of the peculiar phase diagram of the ^3^He–^4^He mixture, and in particular of the phase separation appearing below 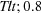 K. This phase separation consists in the coexistence of a concentrated and a diluted phase, the latter having a ^3^He molar fraction of *x* = 0.063 when extrapolated to *T* = 0. The enthalpy difference of the two phases leads to a net cooling by transferring ^3^He from the concentrated phase to the diluted and mostly ^4^He-containing phase. For a standard ^3^He–^4^He dilution refrigerator, *T*_lim_ ≃ 10 mK (Pobell, 1992 ▸). The basic principles of ^3^He–^4^He dilution and its use as a refrigerator for XAS measurements are reported in detail by Sainctavit & Kappler (2001 ▸) and Letard *et al.* (2007 ▸).

Three different research teams (Laboratoire pour l’Utilization du Rayonnement Electromagnétique, LURE; IPCMS; IMPMC) collaborated to develop a first version of a ^3^He–^4^He refrigerator dedicated to XMCD measurements and successfully used during the last 15 years on different European synchrotron-radiation centers (Sainctavit & Kappler, 2001 ▸; Letard *et al.*, 2007 ▸), particularly in the field of molecular magnetism (Mannini *et al.*, 2009 ▸).

Recently, the setting up of the DiLux Consortium of ten European laboratories allowed us to propose a new project of ULT equipment on the DEIMOS beamline at SOLEIL.

The present paper deals with the development of a new ^3^He–^4^He dilution refrigerator, working under ultrahigh-vacuum (UHV) conditions with significant gain in performance compared with the previous version, namely a lower temperature limit (*T*_lim_ = 220 mK), an optimized thermal shielding, the reduction of the eddy current power, easier cool-down and warm-up procedures and user-friendly management of the experiments. Furthermore, the installation of this system in the DEIMOS beamline allows the possibility of *in situ* sample transfer to/from additional UHV chambers and a glove box, providing possibilities for specific *in situ* sample preparation (ion sputtering, temperature annealing up to 1000°C, metal and organic layer deposition) and characterization (scanning tunneling microscopy, electron diffraction, Auger spectroscopy). For these *in situ* prepared samples, special holders have been developed that allow us to reach sample temperatures very close to *T*_lim_.

To illustrate the performances of the new device, we describe here two XMCD experiments as a function of temperature (0.3–4.2 K) and magnetic field ( ± 3 T cycle). As a first example, we illustrate the XMCD at the Er-*M*_4,5_ edges in the paramagnetic Er_0.025_Pd_0.975_ alloy; then, by recording the Fe-*L*_2,3_ edges, we probe the metastable behavior of a Fe_4_ single-molecule magnet (SMM) chemisorbed as a monolayer on Au(111).

## 2. Description of the specificities of the ULT setup   

This section describes the different pre-requisites for performing ULT-XAS experiments with soft X-rays. Dilution refrigerators are of quite widespread use when measuring magnetization, transport properties or neutron diffraction. However, XMCD measurements impose several constraints, such as a UHV environment, a specific pre-cooling of the insert, an electrical sample insulation for the total electron yield (TEY) detection mode and a shielding of the parasitic infrared (IR) radiation inherent to the optical access.

### 2.1. Cryomagnet   

**Figure 1:**
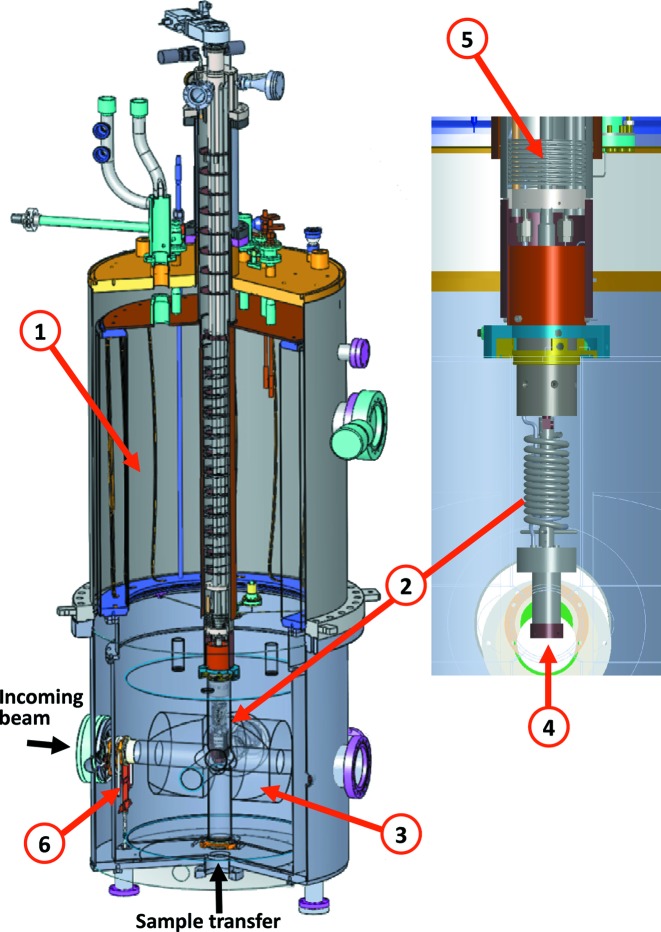
CAD of the cryostat: (1) main He_liq_ tank, (2) ^3^He–^4^He dilution refrigerator, (3) cryomagnet, (4) sample, (5) 4 K tank used for pre-cooling and (6) 4 K thermal retractable shield. Black arrows: the sample-transfer and incoming beam axes. The total height is around 200 cm and the diameter is 60 cm. The diameter of the bottom part of the refrigerator is ∼50 mm.

The new insert has been implemented in the existing cryo-magnet of the DEIMOS beamline (Ohresser *et al.*, 2014 ▸), comprising two superconductive magnets delivering magnetic fields up to 7 T and 2 T, along and perpendicular to the X-ray beam, respectively. The maximum sweeping rate is 0.05 T s^−1^. The typical base pressure is in the low 10^−10^ mbar range without any mechanical pump (a possible source of vibrations and noise) owing to the efficient cryogenic pumping. The dilution refrigerator is mounted in the vertical bore of the cryomagnet. The sample is centered in a six-flange cross on a vertical (*z* axis) translator, having an additional angular degree of freedom θ around the *z* axis. A motorized linear motion, perpendicular to the *z* axis and the X-ray beam, is obtained by translating the whole chamber with respect to the X-ray beam (Ohresser *et al.*, 2014 ▸). A computer-aided design (CAD) of the cryostat with the dilution refrigerator insert is given in Fig. 1 ▸.

### 2.2. Dilution refrigerator-UHV environment   

The ^3^He–^4^He dilution refrigerator has been manufactured by the company CryoConcept (4 avenue des Andes, 91952 ▸ Courtaboeuf, France, http://cryoconcept.com/). The refrigerator benefits from a Joule–Thomson expansion stage, which avoids the need for a 1 K tank and is exclusively built out of UHV-compatible materials in order to avoid pollution of the vacuum and to reach ultimate pressures. Stainless steel (grade 316 L), Cu (Cu-OF) and Cu:Be alloys have been privileged and the standard wire-tin soldering has been replaced by UHV conductive epoxy gluing. This allows us to keep a pressure in the low 10^−10^ mbar range or below after a gentle bake-out.

### 2.3. Pre-cooling of the refrigerator   

**Figure 2:**
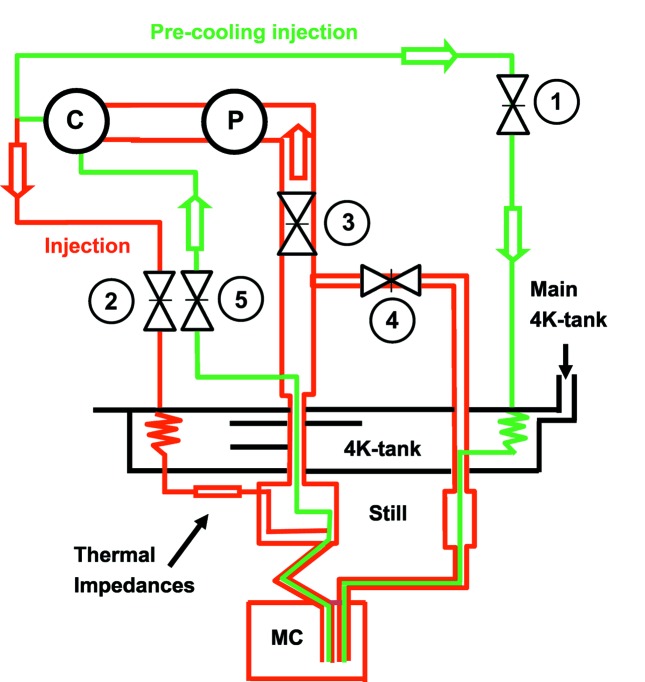
Scheme of the refrigerator circuits (Courtesy of CryoConcept). Green: pre-cooling mode. Red: dilution mode. C = compressor, P = pump and MC = mixing chamber. Black: 4 K-tank of the refrigerator, fed by the ^4^He main reservoir. During the pre-cooling mode, the valve positions are: (1) and (5) = open; (2), (3) and (4) = closed; P = off and C = on. For the dilution mode, the valve positions are: (1) and (5) = closed; (2) and (3) = open; P = on, C = on; and (4) = open only for the pre-cooling circuit purging.

**Figure 3:**
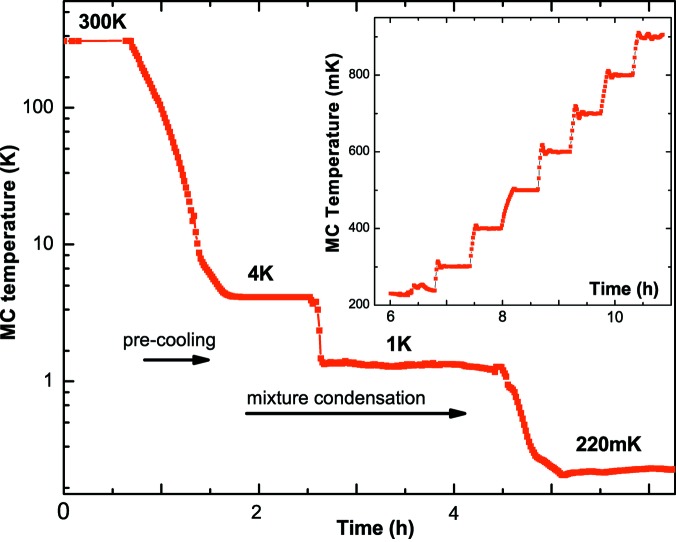
Evolution of the MC temperature on a logarithmic scale as a function of time during the pre-cooling and mixture-condensation procedures. Starting from 300 K, about 4 h are required to reach *T*_lim_. The inset illustrates the temperature stabilization at various set points, from 200 mK to 900 mK, every 100 mK.

For a standard-dilution refrigerator, the pre-cooling procedure, which utilizes an exchange gas, takes a few minutes. As the current insert cannot be cooled down by this method, a secondary He mixture circuit which bypasses the thermal impedances is used. A small fraction (10%) of the total He mixture is injected into this bypass circuit, cooled down by the 4.2 K tank of the variable temperature insert that is fed by the main He reservoir through a capillary and injected directly into the mixing chamber (MC) (Fig. 2 ▸). The bypass circuit is purged when the MC temperature reaches about 4 K, and then the condensation process starts. This pre-cooling procedure speeds up the cool-down process by a factor of five compared with our previous version. The MC cool-down from 300 K to 4.2 K lasts less than 45 min (see Fig. 3 ▸).

### 2.4. Mixing chamber – TEY detection   

The MC located at the bottom part of the refrigerator is the crucial part of a dilution fridge in terms of performance. The sample holder is attached (screwed in/out) to the MC to ensure excellent thermal contact, which is mandatory to reach an optimal thermal conduction. Furthermore, low-noise TEY detection requires an electrical insulation of the sample holder larger than 100 GΩ.

#### 2.4.1. Electrical insulation of the MC   

Using the experience of our previous version, we built the MC as an assembly of a top and a lower metal part, separated by a sapphire ring which ensures a quasi-perfect electrical insulation (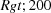 GΩ) of the lower part of the MC, and, correlatively, a high sensitivity of the sample current measurement (a fraction of fA). This technique provides a perfect sealing whatever the temperature, with a He leak rate lower than 3 × 10^−10^ mbar L s^−1^. The sapphire ring also ensures an excellent thermal conduction since the hole through the sapphire permits direct contact of the cooling He mixture with the bottom of the MC where the sample holder is screwed. For more details about the mixing chamber, see the work by Letard *et al.* (2007 ▸).

#### 2.4.2. Sample current measurement   

In the soft X-ray range, XAS is most conveniently detected in the TEY mode by measuring the sample drain current (Ebel, 2004 ▸). The electrometer is electrically connected to the MC by a pair of twisted Kapton^®^-isolated manganin wires, one for grounding and the second for sample current measurement. A typical measurement with low flux (10^9^ photons s^−1^, at *E* = 1000 eV, to avoid beam damage on the sample) yields a sample current of about 10 pA, with a signal-to-noise ratio in the 10^4^ range. Note that fluorescence yield (FY) or transmission measurements are also practicable, but present some inconveniences with respect to ULT; FY requires a detector very close to the sample and therefore well thermalized; transmission requires working with ultra-thin samples, which might be difficult to thermalize. In the presence of an external magnetic field, partial electron yield (channeltron, electron analyzers, *etc.*) cannot be used efficiently, so that TEY is in most cases preferred for XMCD.

### 2.5. Radiative heating – limit temperature   

The limit temperature *T*_lim_ depends on many factors such as the parasitic IR radiations, the nominal cooling power of the dilution refrigerator and the incoming X-ray beam power.

#### 2.5.1. Thermal shielding – infrared radiations   

Different thermal shields were placed in the open bores of the cryomagnet:

(i) At the beam entry there is a 4 K thermalized high-purity Al foil (thickness = 0.7 µm). This foil reduces the flux of the incoming beam at the Fe *L*_2,3_-edge energies by a factor of two.

(ii) Along the transfer and back bores there are two retractable IR radiation-proof Cu shields thermalized on the cryomagnet (measured temperature ≃ 15 K).

(iii) The two other bores, perpendicular to the beam axis in the horizontal plane, are dedicated to the lighting and the observation of the sample during sample transfer. They are both shielded by a first sapphire disk placed on the 77 K shield, then a low-pass IR Schott filter followed by a second sapphire disk, both fixed on the 4.2 K canister.

(iv) All bores, except for the upper vertical bore of the cryomagnet, are also equipped with black-painted Cu tubes which are very efficient in reducing IR radiation reflections.

#### 2.5.2. Cooling power of the ^3^He–^4^He refrigerator   

The preliminary tests of the dilution refrigerator insert into a closed cryostat, without any IR radiation on the MC, led to *T*_lim_ = 60 mK, with a measured cooling power of 50 µW at 100 mK and 170 µW at 200 mK. When placed in the cryomagnet, the refrigerator yields a limit temperature of *T*_lim_ = 220 mK. In other words, the parasitic radiation heating is about 200 µW, mostly coming from the IR radiations through the different bores, and especially from the one above the cryomagnet which could be the main limiting cause. Note that *T*_lim_ of our previous version was 500 mK, as determined through the relaxation time of an SMM (Mannini *et al.*, 2009 ▸; Klar *et al.*, 2014 ▸). In this device, the temperature is measured with a full range thermometer (CryoConcept homemade, radiation protected sensor) inserted in the MC, with a high sensitivity between 10 mK and 350 K regardless of the temperature domain. It has been calibrated at ULT by a reference RuO_2_ sensor mounted at the sample position, which implies by construction that *T*_MC_ = *T*_sample_. This calibration indeed includes the thermal impedances between the helium mixture and the sample. The precision of the sensor is below 1 mK and the uncertainty of the absolute sample temperature will depend on the sample itself and how it is fixed. For the measured samples we estimate it to be about ±25 mK.

#### 2.5.3. Power of the incoming X-ray beam   

A flux of 10^11^ photons s^−1^ (at *E* = 1000 eV) corresponds to *P* = 16 µW, which is much lower than the refrigerator power of 50 µW at 100 mK. For these experiments a much lower flux has been used in order to avoid beam damage. Upon X-ray irradiation of about 10^9^ photons s^−1^, the measured temperature at *T*_lim_ increases by only 5 mK with an incoming X-ray beam around 1000 eV.

### 2.6. Sample transfer – experimental procedures   

For sample transfer, the topmost part of the refrigerator is maintained at low temperature in order to avoid an excessive boil-off of the main He liquid tank. The bottom part of the MC is equipped with a heater resistor which permits the MC warm-up to *T* = 300 K for sample transfer; the sample holder, which is at room temperature, is then screwed onto the MC. This solution is more convenient compared with a cold sample transfer (Beeck *et al.*, 2016 ▸), since it does not require an additional cold finger to pre-cool the sample. Another novelty of this new setup is that thermal stabilization, within a few percent, is achieved over a wide range of temperatures (220 mK–350 K). The refrigerator can maintain a set-point over a period of 1 day or more. Furthermore, the temperature remains stable during the He refill of the reservoir of the superconducting coil, even if the sample is at ULT. All the temperature control is automatic and one needs only to define the temperature set-point into the piloting software. However, the range between 1.2 K and 4.0 K is not straightforward to reach since it is just in between the dilution mode and the so-called 1 K-pot mode.

Passing from 4.2 K to 300 K (sample transfer) and back to 4.2 K requires about 90 min. Three additional hours are required to reach the ULT domain. Fig. 3 ▸ shows the variation of the MC temperature as a function of time during the pre-cooling and the mixture-injection procedures. These two steps are faster by a factor of at least five, with respect to our previous version, and even more so with respect to other setups (Beeck *et al.*, 2016 ▸). The inset of Fig. 3 ▸ illustrates the stability of temperature regulation at various set-points in the 0.2–1 K range.

### 2.7. Eddy current effects   

The magnetic field sweep induces eddy currents in the MC, the sample holder and the sample itself. The corresponding heating power is proportional to the square of the magnetic-field sweeping rate (d*H*/d*t*)^2^, the electrical conductance and some geometrical factors. To minimize this effect, the sample holder and the MC are built out of a Cu:Be alloy (2% of Be atom) that reduces the conductance in the ULT regime and, correlatively, the heating power of eddy currents, at least by a factor of ten with respect to pure copper.

**Figure 4:**
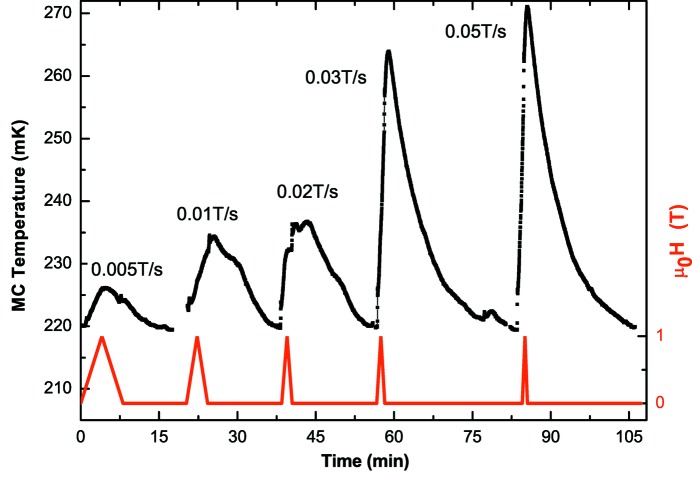
Evolution of the MC temperature for different sweeping rates of the magnetic field. For each rate, the field varies continuously from 0 T to 1 T and back to 0 T (red lines).

In order to find an optimal field sweeping rate, the evolution of the MC temperature was studied for different sweeping rates. For each rate, the field was varied continuously from 0 T to 1 T and back to 0 T (Fig. 4 ▸). For a 0.02 T s^−1^ sweeping rate, the temperature increase is below 20 mK, with a return to the initial temperature in 15 min. For the continuous magnetic field variations when measuring XMCD-detected magnetization curves, the sweeping rate is fixed to 0.01 T s^−1^, leading to a temperature increase of about 50 mK in the case of a magnetic loop amplitude of ±3 T.

## 3. First experimental results obtained with the new setup   

In order to check the ULT device performances we analyzed two different magnetic phenomena showing a strong temperature dependence: first, a paramagnetic Er_0.025_Pd_0.975_ alloy, and, second, a monolayer deposit of a SMM belonging to the widely investigated class of Fe_4_ molecules, which present a temperature-dependent magnetic hysteresis below 1 K. XMCD experiments on the ErPd alloy and SMM layer were performed at the Er *M*_4,5_- and Fe *L*_2,3_-edges, respectively.

### 3.1. Er_*x*_Pd_1−*x*_ alloy   

The solubility of Er in f.c.c. Pd is about 10 at% (Loebich & Raub, 1973 ▸), and the paramagnetic state of Er atoms in such alloys persists down to 400 mK. Therefore, they can be used as *in situ* thermometers. When *x* ≤ 0.1, the Er_*x*_Pd_1−*x*_ alloy develops a magnetically ordered state at temperatures lower than a critical value *T*_o_, which is *x*-dependent. For *x* = 0.1, *T*_o_ ≃ 0.4 K (Delobbe, 1999 ▸; Paulsen, 1999 ▸), hence we chose *x* = 0.025 as a compromise between lowering *T*_o_ as much as possible and ensuring a sufficiently large magnetic signal. Supposing a linear variation of *T*_o_ with *x*, we expect *T*_o_ ≃ 100 mK for *x* = 0.025. Indeed, for *x* = 0.025 and in a low magnetic field (μ_0_ *H* = 9 mT), we did not observe any magnetic coupling for temperatures above 200 mK.

#### 3.1.1. Sample preparation, X-ray diffraction characterization and magnetic properties   

The alloy was prepared by triarc melting of the appropriate amounts of metals under a purified argon atmosphere, using a homemade water-cooled Cu plate and non-consumable thoriated W electrodes. The purities of the starting materials were 99.99% (Pd) and 99.9% (Er). The ingot was remelted ten times and inverted after each melting to promote mixing. The as-cast Er_0.025_Pd_0.975_ alloy was homogenized at 900°C for 24 h in a sealed silica tube and then water quenched.

The crystalline phase of the sample was determined using a Bruker D8 Advance diffractometer equipped with a LynxEye detector at the monochromatic wavelength of Cu *K*α_1_, λ = 1.54056 Å. The expected f.c.c. phase was confirmed (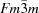 space group), with the lattice parameter *a*_ErPd_ = 3.9052 ± 0.0024 Å at 300 K, as compared with the pure Pd metal, *a*_Pd_ = 3.8921 ± 0.0017 Å; this increase of the lattice parameter means that Er actually forms a solid solution with Pd.

SQUID measurements for the Er_0.025_Pd_0.975_ alloy, performed in the 0.1–4.2 K and 0–3 T ranges, serve as a reference of the bulk Er magnetization. In order to sort out the Er magnetic contribution, the total magnetization was corrected by subtracting the Pd matrix magnetization (Pd metal and extra impurities) measured in the same *T* and μ_0_ *H* ranges.

### 3.2. Er-*M*_4,5_ XAS–XMCD of the ErPd alloy   

For the XAS–XMCD measurements, the sample was fixed with Cu plates screwed on the sample holder in order to ensure the best thermal conduction with the MC. In a UHV chamber connected to the main chamber of the cryo-magnet, the surface of the sample has been scraped with a rotative diamond file for cleaning in a vacuum of *P* ≃ 2 × 10^−9^ mbar, then immediately transferred into the measurement chamber and screwed onto the refrigerator MC.

Two series of experiments were performed: XMCD isotherms as a function of magnetic field and XMCD measurements at different temperatures in a constant magnetic field. Note that the experiments are limited to 300 mK since the Schott filters were implemented afterwards.

**Figure 5:**
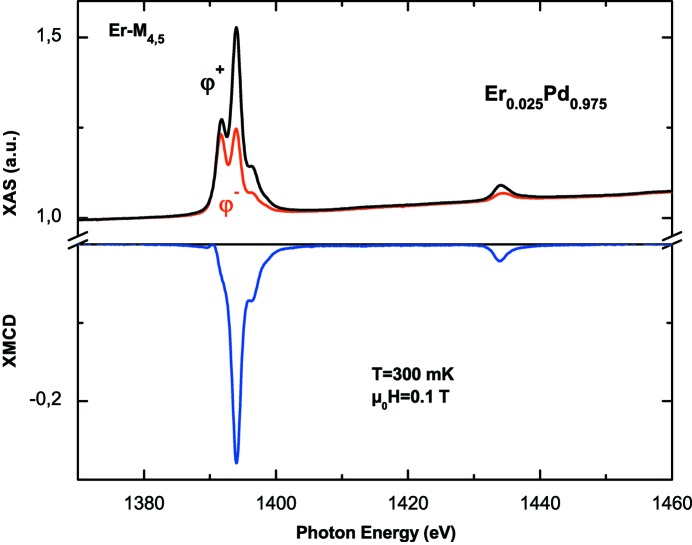
XAS and XMCD at the Er *M*_4,5_-edges in the Er_0.025_Pd_0.975_ alloy at *T* = 300 mK and μ_0_ *H* = 0.1 T. The result is extracted from eight XAS spectra, following the φ^+^, φ^−^, φ^−^ and φ^+^ light-polarization sequence for two opposite magnetic field directions.

Fig. 5 ▸ gives an example of XAS and XMCD at the Er *M*_4,5_-edges for the Er_0.025_Pd_0.975_ alloy at *T* = 300 mK and for μ_0_ *H* = 0.1 T. The spectra were recorded by using the fast continuous-energy scan mode (Joly *et al.*, 2016 ▸), which lasts about 2–3 min for a spectrum width of about 100 eV, with a remarkable signal-to-noise ratio of >10^4^, a photon flux of 1.5 × 10^9^ photons s^−1^ at 1400 eV and a beam size of 0.8 mm × 0.8 mm.

As expected for a pure *J* state, the different structures observed in the XAS spectra correspond to those calculated with a ligand–field multiplet model for an Er^3+^ ion with *J* = 15/2, *L* = 6 and *S* = 3/2 (Goedkoop *et al.*, 1988 ▸). The spectral signatures, *i.e.* the multiplet structure of both XAS and XMCD, are independent of temperature, external magnetic field and electrical crystal field (Schillé *et al.*, 1993 ▸); only the intensity can change.

#### 3.2.1. XMCD *versus* magnetic field   

**Figure 6:**
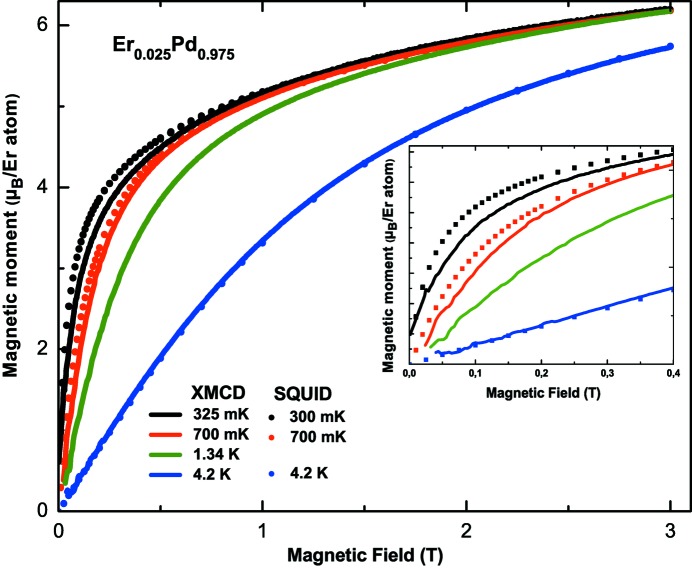
Magnetic characterization of the Er_0.025_Pd_0.975_ alloy: XMCD isotherms (continuous lines), scaled to the 4.2 K magnetization, *versus* magnetic field (μ_0_ *H* = 0–3 T) in the 0.3–4.2 K range. Magnetic isotherms (•–•) from SQUID measurements in the same temperature range. The inset presents an enlargement of the low-field region up to 0.4 T.

Fig. 6 ▸ reports the XMCD and SQUID-detected magnetization curves measured between 0.3 K and 4.2 K for μ_0_ *H* ≤ 3 T. The XMCD magnetization curves are obtained in a fixed-energy mode. The TEY signal is recorded as a function of the magnetic field (±3 T) with a constant sweeping rate (0.01 T s^−1^), first at the energy of the maximum XMCD intensity (1394 eV, Er *M*_5_-edge) and then at the pre-edge (1384 eV) for the two circular light polarizations (∼90 min). The presented XMCD isotherms are the result of the averaging of four curves; the negative-field branch has been symmetrized and averaged with the positive-field branch.

Since the SQUID and XMCD magnetization curves should yield the same behavior as a function of field, we scaled the SQUID and XMCD results for μ_0_ *H* = 3 T and 4.2 K and applied the same scaling factor to all other XMCD curves. In doing so, the XMCD-detected magnetization curves for all temperatures and all magnetic fields are automatically expressed in Bohr magnetons. We noted some small shifts between XMCD and SQUID data for low magnetic fields; they are caused by a temperature increase during the ±3 T cycles because of eddy current heating. For example, for an initial *T* = 300 mK, the sample temperature oscillates upon field sweep around 325 ± 25 mK. For 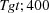 mK, the cooling power of the device is sufficient to compensate the eddy current heating and this temperature drift no longer appears.

Knowing that the TEY detection mode probes about the first 5 nm to 10 nm of the sample surface, we checked the validity of the cleaning procedure by comparing the integrated XMCD intensity as a function of magnetic field in the 2–4 K range with that of Er_2_O_3_. Erbium(III) oxide is an antiferromagnet with *T*_*N*_ = 3.3 K (Narang *et al.*, 2014 ▸) and is the most probable oxidation product that might form at the surface of the ErPd alloy. The magnetization of Er_2_O_3_ as a function of external field is almost independent of temperature in the 2–4 K range, contrary to our XMCD detected isotherms, that are characteristic of a paramagnetic system (Fig. 6 ▸). We can estimate within the error of the XMCD signal that the sample oxidation, if any, concerns less than 5% of the total amount of Er atoms.

#### 3.2.2. XMCD-integrated intensity and bulk magnetization *versus* temperature   

**Figure 7:**
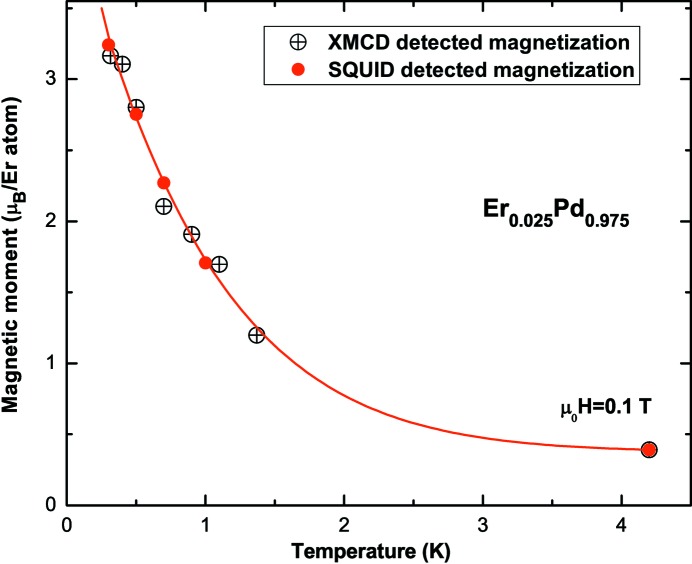
Magnetic moment of Er atoms in the Er_0.025_Pd_0.975_ alloy at μ_0_ *H* = 0.1 T for temperatures varying between 0.3 K and 4.2 K. The red line is a visual guide. Since in a standard-dilution refrigerator the temperature regulation in the 1.2–4 K range is not straightforward (see text), experimental data are missing in this temperature range.

Fig. 7 ▸ presents the magnetization measured by SQUID and XMCD for temperatures varying between 0.3 K and 4.2 K in an external magnetic field of μ_0_ *H* = 0.1 T. Each point of the XMCD intensity is extracted from eight XAS spectra, as explained in the caption of Fig. 5 ▸. The XMCD-integrated intensity is normalized to the magnetic moment at 4.2 K. The good superposition of the two series of measurements, with a maximum deviation of ∼50 mK (Fig. 7 ▸), indicates that the temperature of the XMCD measurement is very close to that recorded by the SQUID. This excellent agreement, much better than that in Fig. 6 ▸, can be attributed to the fact that after each field inversion the temperature was left to stabilize before starting the next measurements, which is indeed impossible for a continuous magnetization curve recording.

It is worth noting that the large variation of magnetization of the Er atoms between 4.2 K and 300 mK (a factor of ∼7) is particularly well suited for using the ErPd alloy as a thermometer. The good agreement between the XMCD and the bulk magnetization at fixed magnetic field down to *T*_lim_ demonstrates that these XAS measurements reveal the bulk properties of the alloy, and that the sample is very well thermalized on the sample holder. The sample temperature is given with confidence by the calibrated thermometer located in the MC.

### 3.3. Investigation of a monolayer of Fe_4_ molecules   

**Figure 8:**
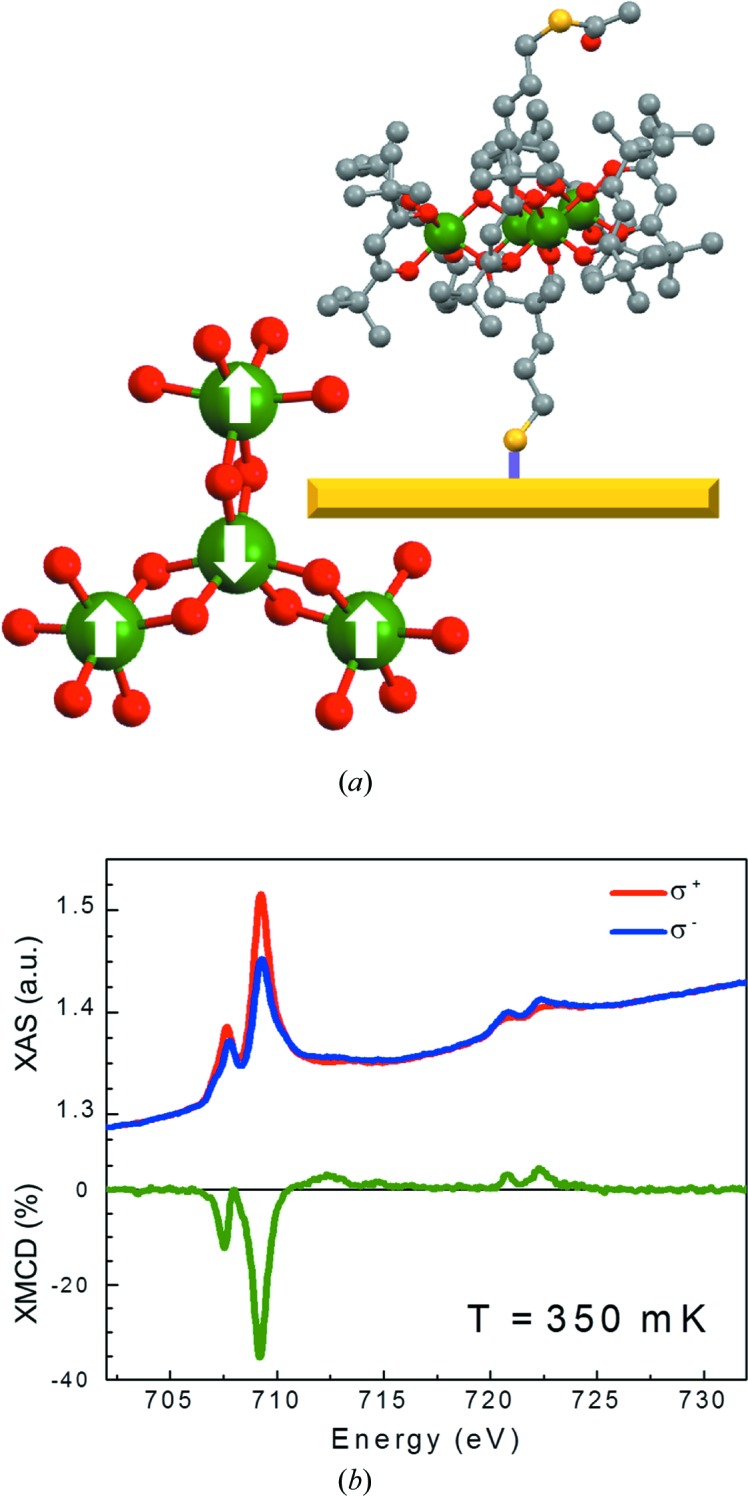
(*a*) Simplified structure of the Fe_4_ complex, highlighting the ferrimagnetic structure in the ground state and representing chemisorption on gold (color code: iron atoms are green, oxygen are red, carbon are black and sulfur are light yellow, hydrogen atoms have been omitted); (*b*) XAS and XMCD spectra of the monolayer of Fe_4_ at 0.3 K under an applied magnetic field of μ_0_ *H* = 3 T.

To further validate the low-temperature performance of this setup, we investigated a molecular sample. Here, we used a monolayer of a tetra-iron (Fe_4_) complex, the archetypal SMM for the realization of hybrid nanostructures (Gatteschi *et al.*, 2006 ▸). The four Fe^3+^ ions (*S* = 5/2, high spin) adopt a metal-centered triangular topology in the structure (as shown in Fig. 8 ▸ *a*). An antiferromagnetic interaction between the central spin and the peripheral ones is present, giving a ground state with a total spin of *S* = 5, schematized by the arrows in Fig. 8 ▸(*a*). They can be chemisorbed onto surfaces, maintaining almost intact their unique low-temperature behavior (Mannini *et al.*, 2009 ▸, 2010 ▸; Cini *et al.*, 2018 ▸). Below 1 K, it is possible to observe a magnetic bistability (*i.e.* the opening of a hysteresis in the magnetization cycle) as a result of the slowing down of the thermally activated process to overcome the anisotropy barrier. The low-temperature behavior is further enriched by the quantum tunneling of the magnetization (QTM), which occurs whenever the quantized spin levels of the molecules are brought into resonance by an external magnetic field (Gatteschi *et al.*, 2006 ▸).

This class of samples represents a valuable benchmark for an ultralow-temperature device, requiring at the same time a sub-Kelvin temperature range and an extreme sensitivity under a very low dose of photons. In fact, these samples (Totaro *et al.*, 2014 ▸) are characterized by a very low concentration of adsorbing atoms (about 2–3 Fe atoms per nm^2^). On the other hand, a strongly attenuated and defocused beam must be used to avoid radiation damage. For these experiments, the photon flux was ∼1.5 × 10^9^ photons s^−1^ at 700 eV and a beam size of 0.8 mm × 0.8 mm. Such a low photon flux thus imposes a strong optimization of drain-current detection in the pA range.

For these tests a novel Fe_4_ derivative has been used, namely Fe_4_(C_3_SAc)_2_(dpm)_6_, where H_3_C_3_SAc, is 5-(acetylthio)-2,2-bis(hydroxymethyl)pentan-1-ol and Hdpm is dipivaloyl­methane. The synthesis and bulk characterization of this compound will be published elsewhere; here we just briefly report on the magnetic characterization of a chemisorbed monolayer prepared following the protocol we have adopted in the past for other Fe_4_ derivatives (Mannini *et al.*, 2009 ▸, 2010 ▸; Totaro *et al.*, 2014 ▸). The purified crystalline material was dissolved in dichloromethane to give a m*M* solution, then a 150 nm flame-annealed polycrystalline Au substrate grown on mica was incubated in the solution. A monolayer deposit was achieved removing the excess of physisorbed material by several washing cycles with pure dichloromethane (Mannini *et al.*, 2010 ▸). The preparation of the sample was carried out in a glove box unit filled with argon gas and directly connected to the DEIMOS beamline.

XAS/XMCD spectra obtained on this sample at 350 mK and μ_0_ *H* = 3 T (Fig. 8 ▸ *b*) provide evidence of the expected spectral features (Mannini *et al.*, 2009 ▸). This confirms the capability of the ULT setup to operate in the required low-photon-flux regime to investigate fragile molecular systems. The intensity of the XAS signal near the *L*_3_-edge with respect to the background (edge jump ∼10%) is consistent with the presence of a monolayer deposit (Totaro *et al.*, 2014 ▸).

**Figure 9:**
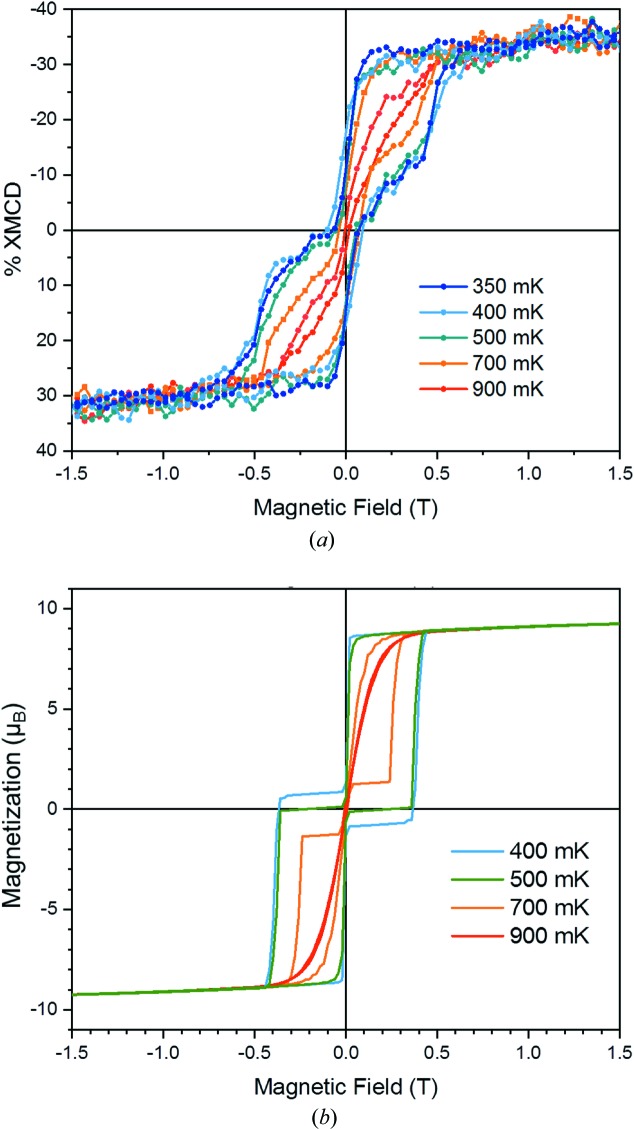
Magnetic characterization of a monolayer of Fe_4_ molecules: (*a*) XMCD-detected magnetization curves (±1.5 T, 0.01 T s^−1^) in the 350–900 mK range; (*b*) simulated hysteresis loops assuming that the easy axis of the molecules forms an angle of 30° with the external magnetic field.

More importantly, Fig. 9 ▸(*a*) shows the temperature dependence of the maximum of the dichroism at the Fe *L*_3_-edges as a function of the magnetic field. These data portray the typical magnetic behavior of Fe_4_ systems, whose hysteresis loops are open below 1 K and become wider with decreasing temperature (Mannini *et al.*, 2010 ▸). The hysteresis curves are almost temperature independent below 0.5 K, indicating the onset of a pure quantum-tunneling regime. Resonant QTM is also responsible for the magnetization steps at 0 and ±0.5 T. Such steps are clearly visible here because of the preferential orientation of molecules with their easy axis close to the surface normal.

To confirm that a good temperature control is also achieved at intermediate temperatures, we have simulated the hysteresis cycles (Fig. 9 ▸ *b*) using a quantum master matrix approach that we have developed previously (Mannini *et al.*, 2010 ▸). The steps in the computed hysteresis curves are more pronounced than observed because only one orientation of the molecules and a unique set of magnetic anisotropy parameters were considered in the calculations. Recent synchrotron-Mössbauer experiments (Cini *et al.*, 2018 ▸) have shown that the process of chemisorption leads to a distribution of molecular geometries that has been neglected here.

The results clearly evidence the capability of performing an ULT-XMCD experiment under a continuously scanning magnetic field with a 50 mK temperature resolution. Such an achievement is far from being trivial and opens relevant perspectives for the low-temperature investigation of hybrid magnetic nanostructures and quantum magnetic systems.

## 4. Conclusions   

In this paper we have described a new ULT-XMCD setup installed on the DEIMOS beamline and dedicated to soft X-ray XMCD experiments. Its improved performances compared with our previous setup have been illustrated by measuring two different physical phenomena with a marked temperature dependence. First, we measured the magnetization of paramagnetic Er impurities in a palladium ingot. In the second experiment, we measured the opening of magnetic hysteresis loops in a SMM monolayer. Both experiments demonstrate, unambiguously, that sub-Kelvin XMCD data can be recorded on two very different systems, thus indicating the versatility and the enormous potential of this spectroscopic tool for magnetic studies well beyond diluted paramagnetic systems or surface science. Despite many worldwide attempts on various synchrotron facilities, this device is certainly unique in providing such performances.
